# Tuberculous Epididymitis in an Immunocompetent Indian Male: A Report of a Rare Case

**DOI:** 10.7759/cureus.46340

**Published:** 2023-10-01

**Authors:** Sankalp Yadav

**Affiliations:** 1 Medicine, Shri Madan Lal Khurana Chest Clinic, New Delhi, IND

**Keywords:** rif assay, xpert, cbnaat, fna biopsy, tuberculous epididymitis, epididymis, mtb (mycobacterium tuberculosis)

## Abstract

Genitourinary tuberculosis is a relatively rare type of tuberculosis. Tuberculous epididymitis is an infection of the epididymis due to *Mycobacterium tuberculosis*. This report describes the case of a 32-year-old Indian male who presented with a small lump in his left scrotum. A diagnosis of genitourinary tuberculosis was established with radiometric investigations and the isolation of the bacteria from the cartridge-based nucleic acid amplification test. He was managed conservatively with anti-tuberculous drugs for six months.

## Introduction

Tuberculosis is a public health challenge for the developing world [1]. It affects people of all ages and from all countries [2]. Tuberculosis, however, is treatable and preventable [3]. This bacterial infection can affect any part of the body, even though it primarily manifests in the lungs [2].

About 15-25% of all tuberculosis cases are extrapulmonary [4]. Tuberculosis of the genitourinary tract makes up 20-73% of cases of extrapulmonary tuberculosis. About 20% of these involve epididymis [2]. It is an unusual presentation, which is infrequently reported in young adults, and has the potential to result in infertility. It mostly originates from retrograde extension from the lower urinary tract or hematogenous dissemination. Infertility is frequently brought on by the inflammation and scarring that follow an infection, which alter normal anatomy and clog the drainage system [2].

This report describes the case of a young Indian man with a small lump in his scrotum. Through radiometric techniques and the isolation of the bacteria, a diagnosis was made of genitourinary tuberculosis. He was initiated on a conservative anti-tuberculous treatment.

## Case presentation

A 32-year-old non-diabetic Indian businessman presented with complaints of a painless lump in his left scrotum for one month. The lump was about 1 cm in size and mobile. There was no history of trauma, urethral discharge, or any surgical intervention. There was no fever, night sweats, or any other constitutional symptom of tuberculosis. He had no notable past medical history and only had one partner with whom he engaged in sexual activity. There was no history of smoking, alcoholism, or any substance abuse.

A general examination was suggestive of a hemodynamically stable man with a body mass index of 26 kg/m^2^. There was no icterus, clubbing, cyanosis, pallor, inguinal lymphadenopathy, or edema. Local examination of the scrotum revealed a non-tender, 1 cm left extra-testicular lump; there was no inguinal lymphadenopathy or discharging sinus. The remainder of his physical evaluation including direct per-rectal examinations was inconsequential.

A probable diagnosis of testicular tumor was made with differentials such as infarction, chronic granulomatous disease, and tuberculosis of the genital tract, and he was advised an ultrasound (USG) of the scrotum, color Doppler, a chest radiograph, and magnetic resonance imaging (MRI), along with routine blood investigations and sputum examinations (Table 1).

**Table 1:** Diagnostic workup of the patient HGB: Hemoglobin; MCH: Mean Corpuscular Hemoglobin; MCHC: Mean Corpuscular Hemoglobin Concentration; MCV: Mean Corpuscular Volume; PCV: Packed Cell Volume; RDW: Red Cell Distribution Width; RBC: Red Blood Cell; WBC: White Blood Cell; DLC: Differential Leukocyte Count; ESR: Erythrocyte Sedimentation Rate; ALK PHOS: Alkaline Phosphatase; AST: Aspartate Aminotransferase; ALT: Alanine Aminotransferase; HCV: Hepatitis C Virus; HIV: Human Immunodeficiency Virus; USG: Ultrasonography; Urine R/M: Urine Routine and Microscopy; CBNAAT: Cartridge-Based Nucleic Acid Amplification Test

Test	Result	Reference range
HGB	15.9	11.5-16.0 g/dL
MCH	22.6	27-33 pcg
MCHC	33.2	31-36 g/dL
MCV	90.2	85-100 fl
PCV	47.0	38.3% to 48.6%
RDW	13.6	0-14%
RBC	5.3	4.7 to 6.1 million cells/mcL
WBC	6.4	4.5-12.0 K/uL
DLC		
Neutrophils	38	55-70%
Lymphocytes	45	20-40%
Monocytes	9	2-8%
Eosinophils	6	1-4%
Basophils	2	0-1%
ESR	76.0	0 to 22 mm/hr
Serum sodium	136.0	135-145 mmol/L
Serum potassium	4.5	3.5-5.1 mmol/L
Serum calcium	8.4	8.5-10.5mmol/L
Serum chloride	99.7	98-107 mmol/L
Blood culture	Sterile	Sterile
Serum bilirubin (total)	0.2	0.2-1.0 mg/dL
Serum bilirubin (direct)	0.3	0.2-1.0 mg/dL
Serum bilirubin (indirect)	0.2	0.2-1.0 mg/dL
ALK PHOS	103.0	30-115u/L
Albumin	3.6	3.5-5 g/dl
Serum creatinine	0.58	0.51-0.95 mg/dL
AST	30.0	0-40u/L
ALT	29.0	0-40u/L
Anti-HCV antibodies	Non-reactive	Reactive-Non-reactive
HIV (I and II)	Non-reactive	Reactive-Non-reactive
Fasting blood sugar	90.0	70-99 mg/dL
Activated partial thromboplastin time	32	25-35 seconds
Serum angiotensin-converting enzyme levels	31	<40 nmol/mL/min.
C-reactive proteins	0.4	0.3 to 1.0 mg/dL
Mantoux test	20	0-15 millimetres
Hepatitis B surface antigen	Negative	Negative-Positive
Urine R/M	Unremarkable	Unremarkable-Remarkable
Alpha fetoprotein	10	0-40 ng/mL
beta-HCG	0.6	<2 mIU/mL
Semen analysis	Unremarkable	Unremarkable-Remarkable
Urine culture	Unremarkable	Unremarkable-Remarkable
Sputum smear microscopy	Negative	Negative-Positive for *Mycobacterium tuberculosis*
CBNAAT of induced sputum and urine	Not-detected	*Mycobacterium tuberculosis* Not-detected-Detected

**Figure 1:**
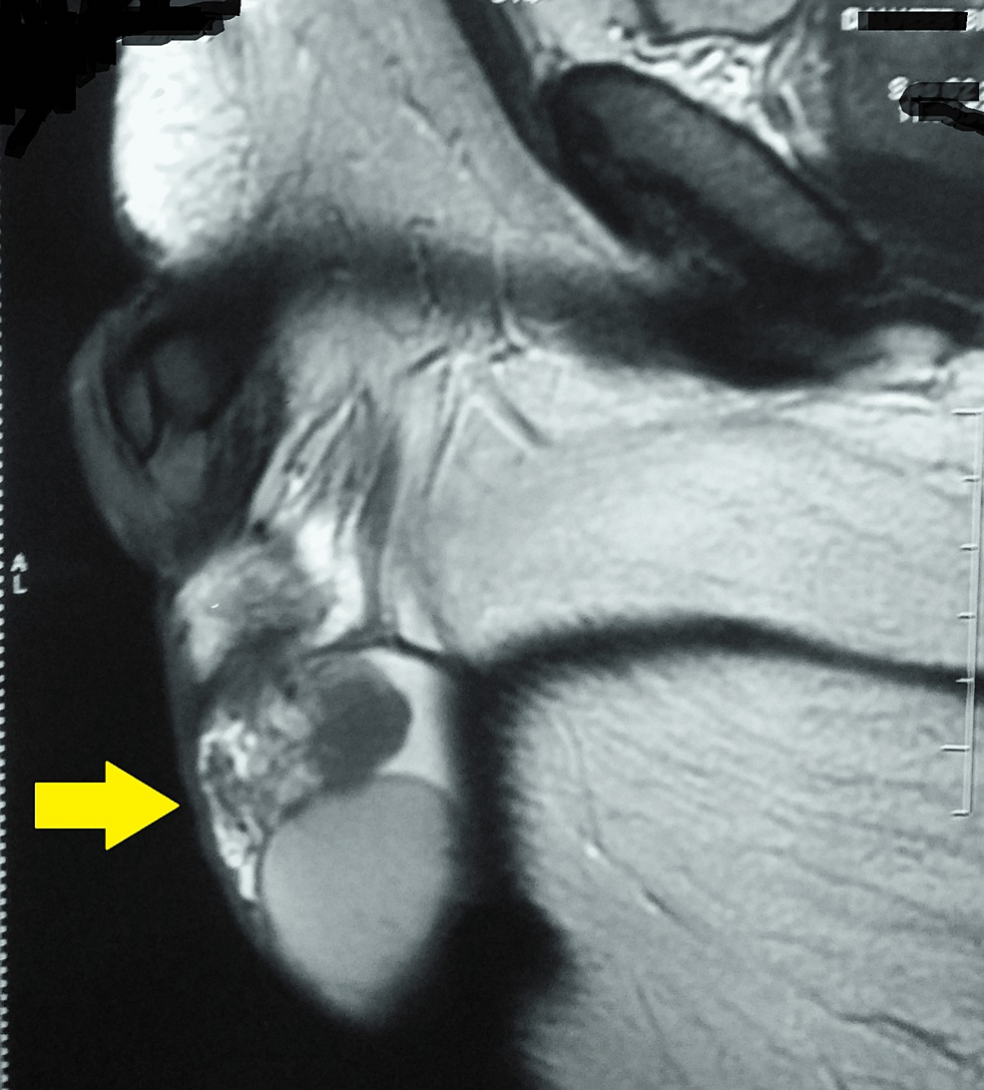
MRI of the scrotum suggestive of nodular thickening in the region of the head of the left epididymis MRI: Magnetic Resonance Imaging

**Figure 2:**
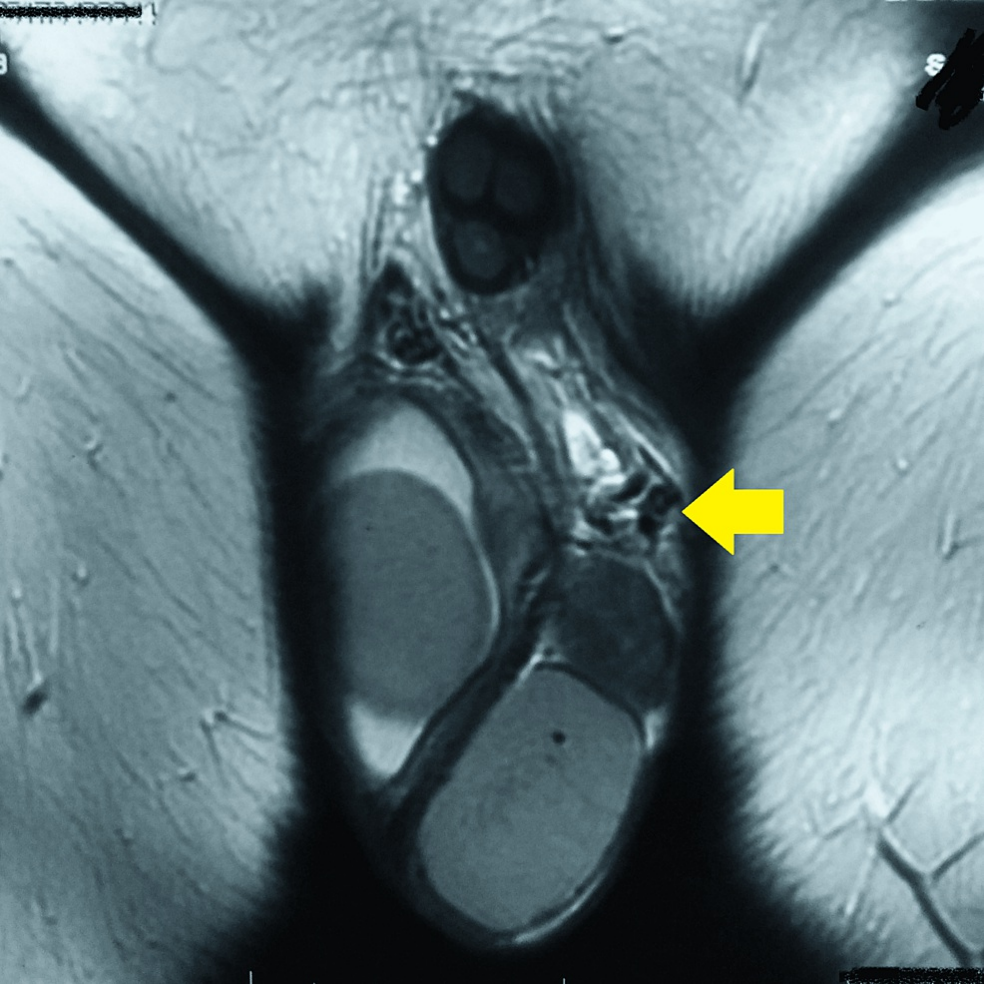
MRI of the scrotum suggestive of nodular thickening in the left epididymis MRI: Magnetic Resonance Imaging

**Figure 3:**
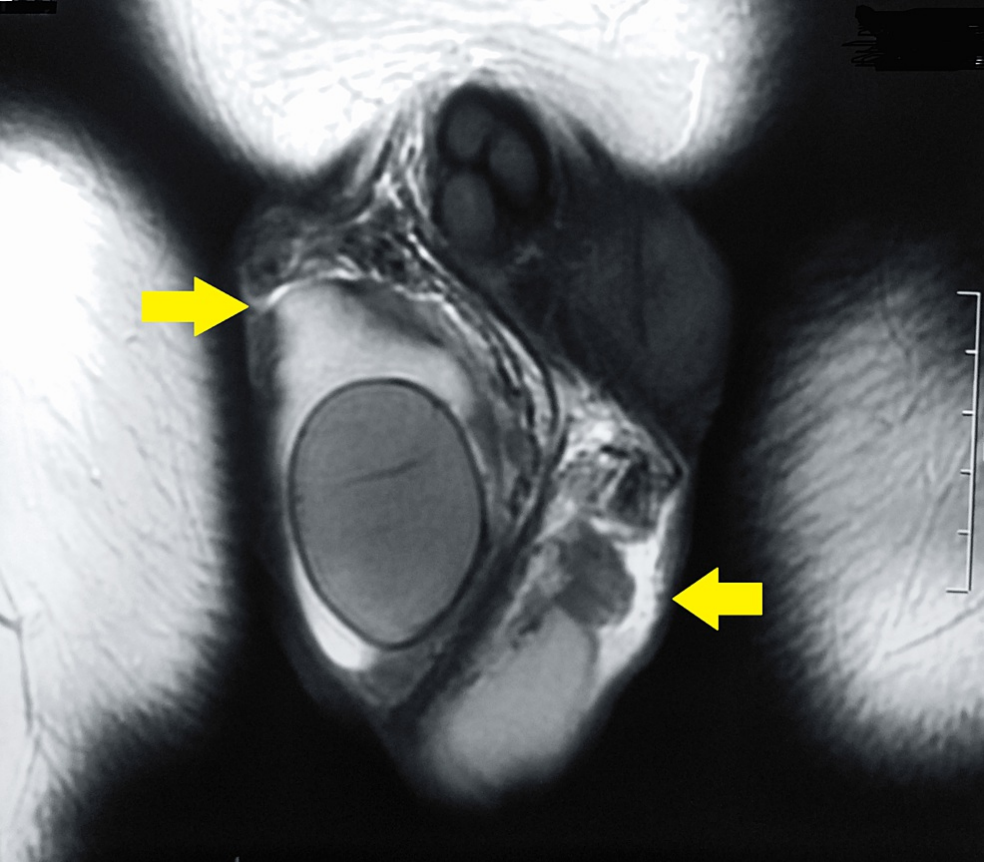
MRI of the scrotum suggestive of bilateral involvement of the epididymis MRI: Magnetic Resonance Imaging

USG of the scrotum revealed a swollen left epididymis and a well-defined, unilateral, spherical, isoechoic lesion measuring 1.0 x 0.96 cm. On color Doppler flows, both testes were reported to be normal. This was followed by MRI of the scrotum, which was suggestive of nodular thickening in the region of the head of the left epididymis, measuring 19.5 x 16.0 x 26.0 mm. Also, the head and proximal body of the right epididymis were thickened by 13 mm. The rest of the structures, including both testes, were normal (Figures 1-3).

**Figure 4:**
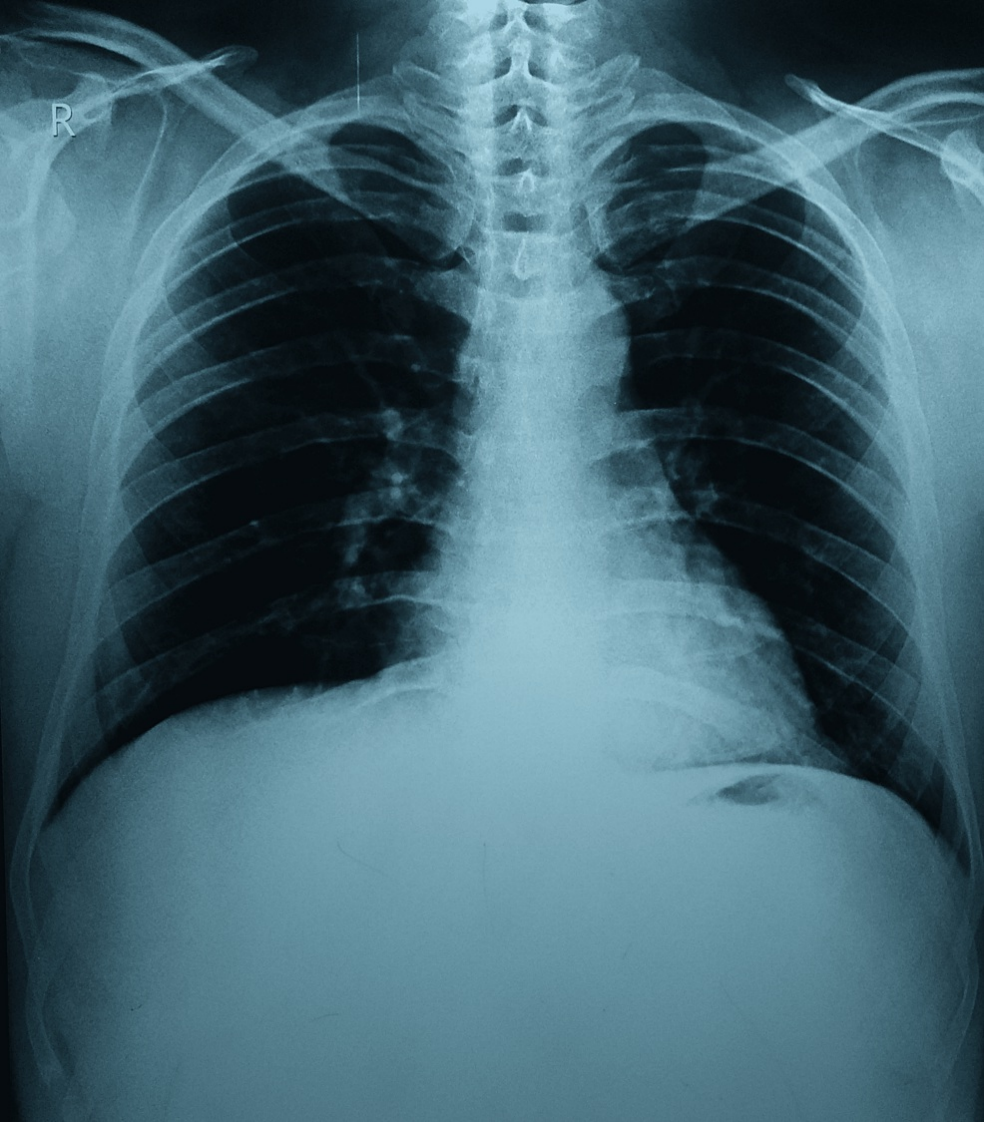
Chest radiograph (P-A view) not suggestive of tuberculosis P-A: Posteroanterior

On the chest radiograph, there was no sign of tuberculosis in the lungs (Figure 4).

Further, a fine needle aspiration biopsy was done from the lump, and samples were analyzed. A cartridge-based nucleic acid amplification test (CBNAAT) of the sample was remarkable for the detection of *Mycobacterium tuberculosis* with no resistance to rifampicin. Histopathology of the biopsied sample showed excessive mixed inflammatory cell infiltration, resulting in ill-defined granulomas with occasional Langhans giant cells. Ziehl-Nielsen staining was non-contributory. Additionally, the line-probe assay and culture were negative.

Anti-tubercular treatment with rifampicin (450 mg), isoniazid (300 mg), ethambutol (800 mg), and pyrazinamide (1500 mg) was initiated for two months before a four-month continuation phase of treatment with rifampicin (450 mg), ethambutol (800 mg), and isoniazid (300 mg). At the one-month follow-up, the patient had a clinical course of gradual improvement with a decrease in scrotal swelling and no adverse reactions to the anti-tubercular drugs.

## Discussion

Tuberculosis of the genitourinary tract is the most common extrapulmonary clinical presentation after lymphatic involvement [5]. Male genital tuberculosis is a rarer form of genitourinary tuberculosis and is mostly found in conjunction with kidney tuberculosis [2]. It is usually reported in the prostate, seminal vesicles, vas deferens, testes, epididymitis, or penis [5]. The epididymis and prostate are the two areas of genital tuberculosis that are most frequently affected [2]. The prostate is the most common site for tuberculosis, and tuberculous infection of the prostate can damage the epididymis by traveling via the vas deferens or through the perivasal lymphatics [6]. Further, tuberculous epididymitis is the most common cause of urological consultations and primarily affects young adults between the ages of 30 and 50 years [7].

All organs may be impacted by hematogenous, lymphatic, or contiguous dissemination of *Mycobacterium tuberculosis*, including the epididymis [7]. Rarely does genital tuberculosis occur alone. Contrary to what was observed in this case, it is more frequently an undesirable consequence of urinary tuberculosis [7]. Further, the cause of infection in this case could be attributed to the endemicity of tuberculosis in India and a probable hematogenous spread of the bacteria to the epididymis.

The primary issue with genital tuberculosis is diagnosis, which is frequently challenging and delayed in the absence of other suggestive sites, an impression of transmission, or a history of tuberculosis [2,7]. Further, due to nonspecific symptoms, persistent protean, cryptic clinical presentations, ambiguous results of imaging techniques, and a lack of awareness among clinicians of the likelihood of tuberculosis, it is a neglected clinical condition and can easily go unnoticed [7]. *Mycobacterium tuberculosis* is seldom incriminated (2-3% of cases of genital tuberculosis only) [7]. In the absence of bacteria in the urine or semen, a biopsy sample of the epididymis is examined histologically to determine the diagnosis with certainty.

Invasive diagnostic techniques, such as computed axial tomography-guided fine needle aspiration biopsy or USG, are frequently necessary to collect biological samples for diagnosis [2]. Although more popular and sophisticated molecular methods for timely *Mycobacteria* DNA detection have emerged recently, reliable microbiological diagnoses still rely on cultures [2].

Delaying treatment leads to oligospermia or azoospermia due to reversible or irreversible organic damage to the genitalia, which affects fertility [7]. Moreover, it could result in psoas abscesses, testicular abscesses, perineal sinuses, liquefaction of granulomas, perforation into the urethra, Addison’s disease, inappropriate antidiuretic hormone secretion, or central nervous system involvement [8].

Management is essentially medical, with anti-tuberculous chemotherapy [7]. The Indian national guidelines suggest treatment for six months with four drugs in the first two months and three drugs for a period of four months [9]. Surgery is usually indicated in extreme cases where there is a diagnostic delay or in suspected cases of tumor [5]. However, superfluous orchiectomies due to challenges in diagnosis and misdiagnosis are also documented [2].

In a study of 187 men, Viswaroop et al. detected tuberculous epididymitis in 54 with a median age of 32 [10]. Another retrospective study by Man et al. detected 47 cases of histologically confirmed tuberculous epididymitis [11]. They inferred that tuberculous epididymitis in its early stages is challenging to diagnose. When clinical features include epididymal beading changes and an ill-defined epididymis-testis border (both clinically and with radiometric techniques), tuberculous epididymitis is likely to have infiltrated adjacent tissues [11].

This case is important due to the paucity of literature related to such presentations. However, this was only one such case, and this emphasizes the importance of large-scale studies from high-burden countries for specifically determining the course of disease, early identification, and timely management.

## Conclusions

A 32-year-old Indian man is described in this report, who presented with a tiny painless lump in his scrotum. Through radiometric studies and the isolation of the bacteria from the CBNAAT, a diagnosis was established. He was treated conservatively with anti-tuberculous treatment. Tuberculous epididymitis is an important cause of genital tract involvement and may be coupled with serious, potentially fatal outcomes. To guarantee a precise diagnosis and course of treatment, adequate knowledge of this entity is crucial.

## References

[1] Haileamlak A (2018). Tuberculosis continued as global challenge though the burden remained high in low-income and high-income countries. Ethiop J Health Sci.

[2] Mohamed Alı A, Doğan A, Ali MA, Çakmak BS (2023). Testicular tuberculosis: two rare case report. Int Med Case Rep J.

[3] (2023). World Health Organization: Tuberculosis. Tuberculosis.

[4] Rodriguez-Takeuchi SY, Renjifo ME, Medina FJ (2019). Extrapulmonary tuberculosis: pathophysiology and imaging findings. Radiographics.

[5] Huang Y, Chen B, Cao D (2021). Surgical management of tuberculous epididymo-orchitis: a retrospective study of 81 cases with long-term follow-up. BMC Infect Dis.

[6] Jacob JT, Nguyen TM, Ray SM (2008). Male genital tuberculosis. Lancet Infect Dis.

[7] Amara C, Keita MA, Sissoko M (2023). Epididymo-testicular tuberculosis: a case report from bamako. Surg Sci.

[8] Skoutelis A, Marangos M, Petsas T, Chionis I, Barbalias G, Bassaris H (2000). Serious complications of tuberculous epididymitis. Infection.

[9] (2023). Training module on extrapulmonary tuberculosis. Training Module on Extrapulmonary Tuberculosis: Standard Treatment Workflow.

[10] Viswaroop BS, Kekre N, Gopalakrishnan G (2005). Isolated tuberculous epididymitis: a review of forty cases. J Postgrad Med.

[11] Man J, Cao L, Dong Z, Tian J, Wang Z, Yang L (2020). Diagnosis and treatment of epididymal tuberculosis: a review of 47 cases. PeerJ.

